# Divergent trends in structural landscape connectivity from historic and potential future grassland conversion in Alberta, Canada

**DOI:** 10.1371/journal.pone.0325729

**Published:** 2025-08-01

**Authors:** Hossam E. Abdel Moniem, Majid Iravani, Tim McAllister, Kim Ominski, Helene H. Wagner

**Affiliations:** 1 Department of Ecology and Evolutionary Biology, University of Toronto, Mississauga, Ontario, Canada; 2 Department of Zoology, Faculty of Science, Suez Canal University, Ismailia, Egypt; 3 Department of Biological Sciences, University of Alberta, Edmonton, Alberta, Canada; 4 Department of Animal Science, University of Manitoba, Winnipeg, Manitoba, Canada; 5 National Centre for Livestock and the Environment, University of Manitoba, Winnipeg, Manitoba, Canada; 6 Lethbridge Research and Development Centre, Agriculture and Agri-Food Canada, Lethbridge, Alberta, Canada; HUN-REN Centre for Ecological Research, HUNGARY

## Abstract

Grasslands across the Canadian prairies are crucial for maintaining biodiversity and ensuring landscape connectivity. In Alberta, a large portion of natural grasslands has been converted to agricultural cropland or other land uses, while the remaining natural grasslands are mainly used as rangeland. However, with increasing crop demand and food security concerns, there is a potential risk of further grassland conversion to cropland, particularly in areas where climate change may enhance suitability for farming. Here, we (1) quantified the impact of the present state of grasslands on maintaining landscape permeability; and (2) determined how the conversion of remaining grasslands to croplands could affect structural landscape connectivity at multiple spatial scales. We simulated four progressive scenarios of grassland conversion to cropland, starting with grasslands identified as most suitable for farming. Our results revealed that structural landscape connectivity, quantified as mean normalized current density with resistance values based on naturalness, decreased by up to 43% in southwestern and central areas of the Parkland and Grassland regions with higher rates of conversion. Conversion scenarios introduced new areas with notably constrained ecological flow in the Grassland region in the southeastern part of the province. Conversely, increased current density was observed in the Rocky Mountain and Boreal regions, which appear to act as alternative pathways for redirected ecological flow. Future grassland conversion is expected to further shift current flow from the grasslands westward through the foothills of the Rocky Mountain and northward into the Parkland and Boreal regions. These findings underscore the critical role of grasslands in maintaining structural landscape connectivity across Alberta, which is essential for supporting biodiversity and gene flow among species. Simulated changes in connectivity were most pronounced at the finer spatial scale, revealing key areas of past and future permeability shifts. Incorporating local land management decisions is crucial for improving landscape permeability and effective connectivity planning province-wide.

## Introduction

Temperate and semi-arid grasslands are recognized as among the most threatened biomes on Earth [1]. In the North American Great Plains, the conversion of grasslands to other land uses, such as cropland, residential or industrial development, has been a progressive threat to biodiversity [2]. The Canadian prairie ecozone, which constitutes 16% of the Great Plains area and spans across the southern parts of the three provinces of Alberta, Saskatchewan, and Manitoba, was once covered by continuous yet heterogeneous natural grasslands [3]. Today, only 25–30% of the original grassland cover within this ecozone remains, primarily in the form of extensive cattle grazing lands [3]. In addition to this loss of grassland habitat, the remaining grasslands today are highly fragmented [4]. Besides being fragmented, the ecological integrity of Alberta’s grasslands has been threatened by agricultural expansion, climate change, and industrial development [5]. Regardless of these stressors, some native grasslands persist with a relatively high biodiversity, and they continue to provide critical habitats for many plant and animal species [6]. Nonetheless, invasive species and habitat fragmentation threatens to further reduce native ecosystems’ capacity to support biodiversity by altering species composition and ecosystem function [7].

In our rapidly changing environment, and especially in light of rising demand for crops combined with the effects of climate change, it is crucial to understand how future land-use changes may impact ecological processes at the landscape scale. Here, we focus on landscape connectivity, which refers to the degree to which a landscape facilitates or impedes the movement of organisms and their genes within and across ecosystems [8,9]. Landscape connectivity determines the dynamics of ecological processes and biodiversity patterns at the landscape scale [8]. Declining landscape connectivity can negatively impact the diversity of living organisms at different levels (from the individual to populations and ecological communities) and, therefore, biodiversity as a whole [10–13]. In addition, climate change can alter the suitability of Alberta’s grassland areas to produce field crops. The land suitability rating system (LSRS), which ranks land by use category based on soil factors, climate factors, and landscape factors, has shown that increasing temperature has the ability to move the areas that are suitable to grow crops such as spring seeded small grains and oilseed crops, into areas that are presently rated not suitable [14]. Lots of this potential will depend on precipitation changes, as some models predict more arid climates which would offset the temperature benefits, by decreasing soil moisture and crop potential. As such lands now classified as less suitable could see the possibility of marginal pressures for production depend on the climate scenario, with the loss of native grasslands impacting biodiversity [15]. Managing landscape connectivity is challenging because it involves different spatial scales and levels of jurisdiction [16]. Landowners typically make decisions about land cultivation at a local spatial scale. However, such local decisions can significantly impact landscape connectivity at broader scales, which are crucial for management and planning at municipal, regional, or provincial levels [17]. Therefore, quantifying landscape connectivity under different land-use scenarios—at scales pertinent to these management levels—is essential for informing decision-making and identifying strategies for land and biodiversity conservation and restoration.

Connectivity assessment over large spatial scales often relies on modelling structural landscape connectivity based on the degree of naturalness without making specific assumptions of species’ behavioural response to landscape structure (i.e., species-agnostic approach) [18–20]. A recent evaluation of structural landscape connectivity throughout North America showed that some of the lowest levels of connectivity occur in Alberta at the interfaces of the Great Plains grasslands, the Canadian Rocky Mountain to the west, and the Boreal region to the north [21]. In these critical areas, much of the original grassland cover has already been lost, and the conversion risk of remaining grasslands has been estimated at greater than 75% [2]. Grasslands in Alberta are key habitats for biodiversity and wildlife, hosting approximately 80% of the listed species at risk and 25% of identified rare native plant species in the province [22]. Despite their significance, Alberta’s grasslands have been undergoing widespread conversion over the past decades [23,24]. Consequently, several species, including a number of large mammals (e.g., bison, grizzly bear, wolf) and multiple grassland birds (a 40% decline in the number of endemic species), have been extirpated from this ecosystem [25]. Numerous conservation projects are being carried out to maintain and improve grassland connectivity in Alberta’s Grassland Natural Region. The Alberta Conservation Association, Alberta Environment and Protected Areas, the Prairie Conservation Forum, and other stakeholders collaborated to create the MULTISAR program which focuses on multi-species conservation at the landscape level [26]. More than 12,000 hectares of endangered grassland have been protected by the conservation efforts of the Nature Conservancy Canada in the southwest. For example, the partnership with the Waldron Ranch Grazing Cooperative demonstrated how biodiversity conservation and sustainable ranching methods can coexist in a way that maintains grassland connectivity [27]. However, little is known about the importance of the remaining grasslands for maintaining landscape connectivity regionally, across the Great Plains, as well as more locally in Alberta. In particular, it is unknown to what degree the remaining structural landscape connectivity at different spatial scales may be threatened by further conversion of Alberta’s grasslands.

This study examines historic and potential future trends for structural landscape connectivity in Alberta at multiple spatial scales via scenario modelling. Specifically, we aim to (1) quantify the impact of the present state of human landscape alteration on structural landscape connectivity compared to an assumed natural reference state; and (2) determine how the potential future conversion of remaining grasslands could impact structural landscape connectivity.

## Materials and methods

### Study area and data

**Fig 1:**
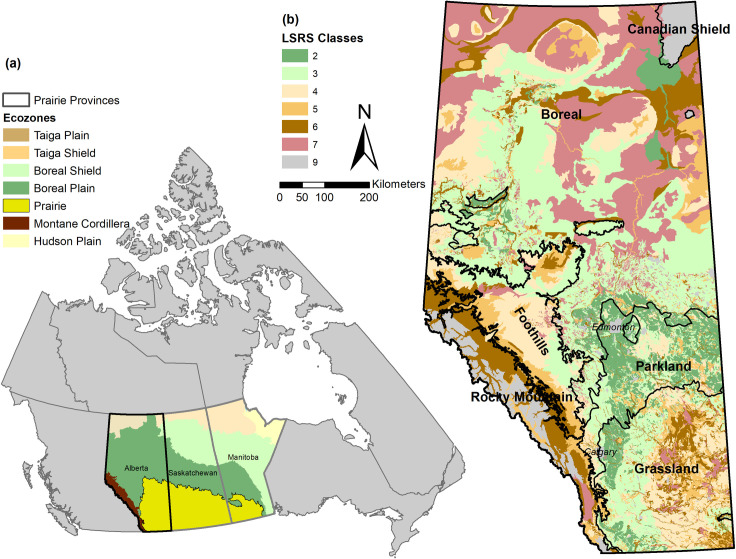
Alberta’s Natural Regions and Land Suitability Rating System (LSRS). **(a)** Map of Canada showing the three Prairie Provinces, Alberta, Saskatchewan, and Manitoba, depicting the prairie region located in the southern part of these provinces. **(b)** Map of Alberta with the 6 Natural Regions and the 8 classes of Land Suitability Rating System (LSRS) ranked based on limitations for cultivation: class-2 (slight limitation), class-3 (moderate), class-4 (severe), class-5 (very severe), class-6 (extremely severe), class-7 (unsuitable), and class-9 (not rated). Data source: Land Suitability Rating System (LSRS), Open Government Licence – Alberta, version 2.1.

The province of Alberta, with a total area of 661,848 km^2^, comprises six Natural Regions (Fig 1): the Boreal region covering a significant portion of northern Alberta, bordered to the northeast by the Canadian Shield region; the Rocky Mountain and Foothills regions to the west; the Grassland region in the south; and the Parkland region, which serves as a transition zone between the grasslands and the forests in the Foothills and Boreal regions (Fig 1). The suitability of land for producing field crops has been classified across these landscapes according to the Canadian Land Suitability Rating System (LSRS). LSRS is a procedure for rating the suitability of land for agricultural spring-seeded small grains and hardy oilseeds based on the soil-climate-landscape potential. It ranges from 1–7 with class 1–3 lands being the most productive, while classes 4–7 are considered to have more limitations for agriculture production. These values are intended to be updated periodically (developed in 1995 and most recently updated in 2019) to inform decisions related to agriculture, development, and energy projects [14,15].

Alberta’s landscapes comprise a dynamic mix of natural environments and various human footprint types, including agriculture, forestry, mining, oil and gas industry, residential and industrial developments, transportation infrastructure, and reservoirs and other anthropogenic water features [28]. We used the Alberta Biodiversity Monitoring Institute’s (ABMI) Human Footprint Inventory (HFI) dataset which comprised 21 polygon-based layers including 115 land-uses divided into six primary categories including agricultural, forestry, transportation, urban, energy, and man-made water bodies. The dataset was derived from the comprehensive 2014 Alberta’s land-use data (for more details on data collection and computation of the land-use geodatabase, see Geospatial Centre ABMI, 2018). The source of this dataset was the Alberta Environment and Sustainable Resource Development, Informatics Branch’s 2014 SPOT6 Satellite Imagery (1.5 m Color SPOT 6 Mosaic) (https://www.alberta.ca/environment-and-parks.aspx).

Alberta’s remaining grasslands (about 6.5 million ha), as determined by the ABMI’s wall-to-wall vegetation layer [29] and the HFI [28], are distributed across several distinct Natural Regions with different climates, soils and vegetation types [30]. However, the majority of the remaining large and relatively intact grassland areas are found in the Grassland region (Fig 1). In addition, Alberta has a long history of cultivating grasslands with perennial forage species, thus converting them into tame pastures. This practice, which primarily supports grazing livestock or produces hay to feed them [31], is particularly common in the productive soils of the Grassland and Parkland regions (Fig 1). For this study, the term grassland encompasses all grasslands, including tame pastures.

### Present-state model of landscape resistance

For the status-quo model (SQ), which reflects the present state of human landscape alteration, we created a landscape resistance map based on the total degree of human modification (*H*) of natural features [20]. First, the vector data for each human-footprint category *j* included in the HFI data for the year 2014 (*k* = 84 categories) was rasterized by assigning a value of either 1 or 0 to each 10 × 10 m cell of a raster covering Alberta: if the cell *i* overlapped with polygons of the human footprint category *j*, a value of *h*_*ij*_ = 1 was assigned; otherwise, a value of *h*_*ij*_ = 0 was assigned. Then, each value *h*_*ij*_ was multiplied by the degree of naturalness value defined for human footprint category *j*, as listed in Marrec et al. [20]. The total degree of human modification *H*_*i*_ of raster cell *I* was determined by the fuzzy algebraic sum [32] of the individual degrees of human modifications *h*_*ij*_ of all categories *j* [18] (Eq 1).


$$\begin{array}{c} H_{i}=1.0-\prod {\mathrm{\backslash nolimits}}_{j=1}^{k}\left(1-h_{ij}\right) \end{array}$$ (1)

We used the method of Theobald [18] and Dickson et al. [33], as modified by Marrec et al. [20], to calculate the human modification index *H* based on two metrics indicative of the degree of modification of natural features from their original state: the degree of physical footprint (*H*_*F*_) and the intensity of human use (*H*_*U*_). Both *H*_*F*_ and *H*_*U*_ were expressed on a scale from 0.0–1.0 (low to high physical footprint or intensity of human use, respectively). These values were assigned through consultation with experts from the Technical Committee of the Alberta Human Footprint Mapping Program [20]. We used these values to calculate an average human modification index (*H*_*FU*_) across both human modification indices [20], where *H*_*FU*_ = (*H*_*F*_ +*H*_*U*_)/2.

We calculated landscape resistance (*R*) as described by Marrec et al. [20] with a modification from Dickson et al. [33]. We rescaled the *H*_*FU*_ values with a scaling function (to create a larger contrast between low and high resistance values) and added additional penalties for resistance due to topography (i.e., slope in percent, *s*) and water bodies *w* in the landscape (Eq 2).


$$R={\left(H_{FU}+1\right)}^{10}+\frac{s}{4}+57.7*w$$ (2)

Thus, the minimum resistance was *R* = 1, and the maximum resistance due to *H*_*FU*_ was (1 + 1)^10^ = 1024. A 100% slope (*s* = 100) on its own led to a resistance value of 100/4 = 25, while for natural water bodies (*w* = 1), the resistance value was 57.7, which corresponded to an intermediate value of *H*_*FU*_ = 0.5: $(0.5+1{)}^{10}=57.7$. The final resistance raster datasets were aggregated to 100 m resolution by calculating the mean from the original 10 m raster to optimize computation time for modelling current density [20].

### Present-state model of current density

We used the software GFLOW [34] to compute an omnidirectional current density map based on the resistance map *R* with 100 m resolution. Specifically, we used circuit theory [35] to simulate the electrical current flow density between all pairs among 50 nodes. The current density value of each raster cell indicates the relative amount of ecological flow expected to pass through this cell. In other words, it quantifies the contribution of each cell to the larger-scale landscape connectivity [34]. Nodes were evenly distributed (~100 km apart) along an outer buffer zone that was 25% larger than Alberta’s boundary polygon to avoid source flow artifacts [36]. To ensure that the same current was emitted from the same node pairs, nodes were not randomly shuffled, and no convergence threshold was used [34]. Instead, the computation continued until all node pairs had been used, and the current density values were summed for each cell across all node pairs. The resulting current density map was clipped to the provincial boundary.

### Modelling scenarios

We repeated the current density modelling described above (status-quo model, SQ) for each of the following historic and future scenarios:

### Null model (NM)

We created this model by assuming a reference state where the entire province was considered natural (i.e., no human footprint; *h* = 0). In this scenario, landscape resistance was only due to natural water bodies and topographic slope assuming that these two features are the only source of resistance to terrestrial organisms’ movement across a natural landscape in absence of human footprint [18].

### Future grassland conversion models (S2–S5)

We integrated both native and cultivated grasslands into four grassland-to-cropland conversion models by considering the LSRS classes 2–5 (Fig 1), examining the grassland areas with the most likely (e.g., LSRS class 2: S2) to the least likely (e.g., LSRS class 5: S5) conversion to annual cropland. Specifically, for each grassland conversion model (S2–S5), we used the corresponding LSRS class to identify and consecutively add converted grassland areas from the less severe (S2) to the extreme conversion scenario (S5). We updated the corresponding degree of naturalness values of converted grasslands from *h* = 0 to *h* = 0.5 before computing an updated resistance map for each scenario. Note that we did not consider the current protection status, i.e., all remaining grasslands falling within a given LSRS class were assumed to be converted to croplands.

### Normalizing current density maps

To ensure all current density maps were comparable, a potential current flow layer was computed by modelling current density for a resistance layer where the resistance value of every raster cell (100 × 100 m) inside Alberta’ ‘s boundary polygon was set to 1. The resistance values in the buffer area were kept constant across all simulations. We then calculated normalized current flow maps by dividing the current density map of each scenario by the potential current flow layer [37]. This method removed any current flow artifacts for the generated maps [21,38,39].

### Current density trends

To quantify changes in current density trends across the Natural Regions of Alberta (Fig 1), we first classified the grid cell values of the normalized current density map created for each grassland conversion scenario using the percentiles of the null model’s (NM) current density distribution as a reference. This resulted in three patterns of the normalized current density: (1) diverted current (top 5 percentile) representing pinch-point areas of high current density values constrained by areas of high resistance; (2) diffused current (middle 90 percentile) representing diffused current in large areas of natural land-covers or otherwise medium-high current density values; and (3) lost or otherwise low (bottom 5 percentile) current density values in more human-modified landscapes (S1 Fig). We tabulated the three classes for each Natural Region and scenario (S1 Table) to identify trajectories across the grassland conversion scenarios (S2–S5).

### Scenario comparisons at different spatial scales

To quantify the contribution of grasslands to structural landscape connectivity, we aggregated the normalized current density values (100 × 100 m resolution) by calculating the mean and standard deviation within spatial units at three scales: (1) Natural Regions (n = 6), (2) municipalities (n = 80), and (3) 65 ha quarter sections (n = 1,022,253). The percent change in mean normalized current density between each grassland conversion scenario (S2–S5) and the status-quo scenario (SQ), and between the status-quo scenario (SQ) and the null model (NM), was then calculated to quantify the loss or gain in connectivity at each spatial unit.

## Results

### Impact of present-state human landscape alteration

Compared to the assumed all-natural reference state (NM), the status-quo model (SQ) resulted in a net decrease in mean normalized current density for the Parkland region (−43.3%), the Grassland region (−15.7%), and the Boreal region (−2.4%) (Table 1). In contrast, the other Natural Regions showed a net increase (Rocky Mountain region: 75%; Foothills region: 34.7%; and Canadian Shield: 6.8%). Given the model assumed that all natural habitats were equal and did not pose additional resistance to movement beyond topography (slope) and natural water bodies—both of which were held constant in all models—an increase in mean current density suggests the presence of pinch point areas, where current is diverted from areas with higher resistance. Therefore, the SQ model indicates a potential diversion of current from the Parkland and Grassland regions to the Rocky Mountain and Foothills regions.

**Table 1:** Normalized current density per Natural Region.

	Conversion scenarios (mean ± SD)					
	*Percent change*					
Natural Region	NM	SQ - NM	S2 - SQ	S3 - SQ	S4 - SQ	S5 - SQ
Grassland	1.42 ± 0.35	1.20 ± 1.15	1.16 ± 1.13	1.06 ± 1.11	0.89 ± 0.99	0.73 ± 0.80
		***−15.7***	***−2.8***	***−11.8***	***−25.7***	***−39.2***
Parkland	1.43 ± 0.39	0.81 ± 0.89	0.77 ± 0.84	0.73 ± 0.79	0.65 ± 0.67	0.59 ± 0.57
		***−43.3***	***−4.7***	***−9.6***	***−19.7***	***−26.9***
Foothills	1.20 ± 0.32	1.62 ± 0.96	1.64 ± 0.97	1.65 ± 0.98	1.67 ± 1.00	1.69 ± 1.01
		***34.7***	***0.9***	***2.0***	***3.2***	***4.1***
Rocky Mountain	0.74 ± 0.32	1.30 ± 0.72	1.32 ± 0.73	1.33 ± 0.75	1.38 ± 0.80	1.39 ± 0.81
		***75.4***	***0.9***	***2.4***	***5.8***	***6.6***
Boreal	1.38 ± 0.43	1.35 ± 0.71	1.35 ± 0.71	1.35 ± 0.72	1.35 ± 0.72	1.35 ± 0.72
		***−2.4***	***−0.1***	***−0.2***	***−0.2***	***−0.2***
Canadian Shield	1.19 ± 0.61	1.27 ± 0.65	1.27 ± 0.65	1.27 ± 0.65	1.27 ± 0.65	1.27 ± 0.65
		***6.8***	***~0.0***	***0.2***	***0.2***	***0.2***

Mean normalized current density and standard deviation (SD) per Natural Region for the null model, NM, the status-quo model, SQ, and four progressive grassland conversion scenarios, S2–S5. Numbers in bold italics indicate the percent change in mean normalized current density between SQ compared to NM, and for each grassland conversion scenario (S2–S5), compared to the status-quo model, SQ scenario.

**Fig 2:**
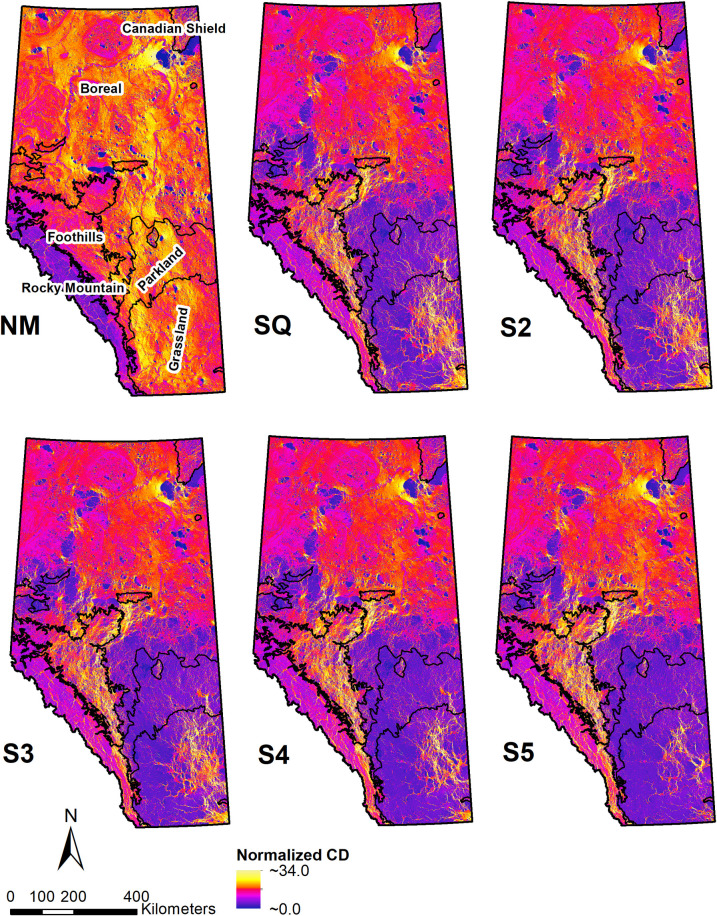
Normalized current density maps of Alberta. 100m resolution maps showing: **NM** is the reference state where current density was modelled based on resistance due to topography and natural water bodies only (no anthropogenic modifications); **SQ** is the present state model where current density was modelled based on resistance due to topography, water bodies, human footprint data for 2014; **S2** is the current density map resulting from the first conversion scenario where all grasslands located within LSRS class-2 were assumed to be converted into cropland, **S3** is the current density map resulting from the second conversion scenario where all grasslands located within LSRS classes 2 and 3 were assumed to be converted into cropland, **S4** is the current density map resulting from the third conversion scenario where all grasslands located within LSRS classes 2–4 were assumed to be converted into cropland; and **S5** is the current density map resulting from the final conversion scenario where all grasslands located within LSRS classes 2–5 were assumed to be converted into cropland. The colour ramp represents raster cells with high current density values in yellow and low current density in purple.

Compared to the NM model, the map of normalized current density for the SQ model revealed a large area of very low current density in the Parkland region and in the western part of the Grassland region (Fig 2). This indicated a widespread loss of ecological flow through these areas. In contrast, the eastern part of the Grassland region exhibited both very low and very high current density values, suggesting that ecological current was concentrated through remaining pathways (pinch points) that followed more natural habitat within an otherwise less permeable landscape. The Foothills region also showed similar pinch points, forming connections either along the foothills of the Rocky Mountain or to the Boreal region. In the Rocky Mountain region, the SQ model indicated a widespread increase in current density (i.e., receiving current diverted from other areas) from a low initial level in the NM model (due to slope), without the emergence of pinch points. Pinch points in the NM model were related to the diversion of currents around lakes.

The visual analysis of Fig 2 helps interpret trends in the proportions of low (“lost current”), intermediate (“diffused current”), and high (“diverted current”) values of normalized current density (Fig 2). In the NM model, which defined the cutoff values for classification, current loss was primarily observed in the Rocky Mountain (due to slope) and the Canadian Shield (due to a high proportion of water bodies) regions. In the SQ model, the Grassland and Parkland regions both exhibited a large proportion of current loss. In the Grassland region, this was accompanied by a significant amount of diverted current (pinch points), indicating the presence of connectivity conduits. The Parkland region showed a much lower proportion of diverted current, suggesting that it lost much of its landscape permeability. Both the Foothills and the Rocky Mountain regions showed a large proportion of diverted current in the SQ model, without a corresponding increase in current loss, indicating that they received current diverted from other regions. Although the Foothills region had more diverted current, the Rocky Mountain region experienced a reduction in the proportion of current loss, as previously shown in Fig 2. The Boreal and Canadian Shield regions also showed both lost and diverted current in the SQ model, nonetheless to a lesser extent compared to the Grassland region.

**Fig 3:**
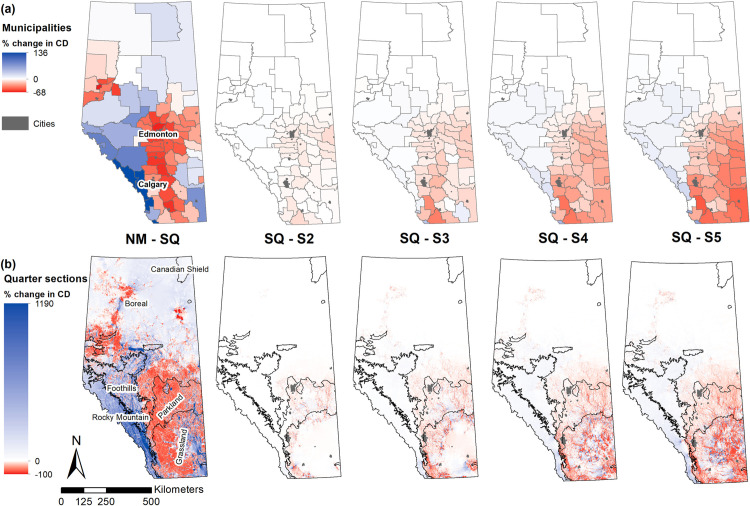
Change in Normalized current density between grassland conversion scenarios. Percent change in mean normalized current density, (a) per municipality, and **(b)**, per quarter section, across grassland conversion scenarios in Alberta. Alberta’s Natural Regions were overlaid on the quarter section maps. NM is the null model where current density was modelled based on resistance due to topography and water bodies only. SQ is the status-quo model where current density was modelled based on resistance due to topography, water bodies, and the average degree of anthropogenic modifications; S2 is the current density map resulting from the first conversion scenario where all grasslands located within LSRS class-2 are simulated to be converted into cropland; S3 is the current density map resulted from simulating the conversion of grasslands located within LSRS class-2 and 3 into cropland, S4 is the current density map resulted from simulating the conversion of grasslands located within LSRS class-2, 3, and 4 into cropland, and S5 is the current density map resulted from simulating the conversion of grasslands located within LSRS classes 2–5 were converted into cropland. Urban municipalities are plotted in dark grey to facilitate orientation.

The map of percent change in mean normalized current density per municipality (Fig 3, a) showed significant variation within Natural Regions and a shift in patterns as grassland conversion scenarios progressed to include soils with very severe limitations for cultivation (S5). The most substantial change in current density occurred historically between the null model (NM) and the status-quo scenario (SQ), ranging from −67.6% to 135.9%. Municipalities in the Parkland region, the western part of the Grassland region, and the central northwest area of the Grand Prairie experienced a notable decline in mean normalized current density. Conversely, the SQ model indicated a considerable increase in mean normalized current density for municipalities in the southern Rocky Mountain region near Calgary, the Foothills region, and the southern Boreal region. See S3 Table for mean normalized current density and standard deviations values per municipality.

At the provincial scale, mean resistance and normalized current density varied among scenario maps at 100 m resolution (S2 Table). The null model (NM) showed the lowest mean resistance (4.757 ± 11.45), and the highest mean normalized current density (1.33 ± 0.44), while the most severe conversion scenario S5 showed the highest resistance (27.59 ± 70.59), and the lowest mean normalized current density (1.23 ± 0.83) among all conversion scenarios.

### Impact of potential future grassland conversion

Overall, the remaining grasslands in the SQ model were concentrated in the Grasslands (387.11 ha) and Parkland (67.53 ha) Natural Regions, with minor areas in the Boreal (29.88 ha), Foothills (1.84 ha), and the Rocky Mountain (8.76 ha) regions (Table 2). These remaining grasslands were distributed across various soil suitability classes (Fig 1). Consequently, the potential future grassland scenarios involved converting a cumulative percentage of total remaining grassland area to crop were: 6.35% for scenario S2, 24.38% for S3, 47.75% for S4, and 69.66% for S5.

**Table 2:** Area of remaining Grassland per Natural Region.

	Remaining Grassland (ha)				
	*% Converted grasslands*				
Natural Region	SQ	S2	S3	S4	S5
Grassland	387.11	372.97	311.46	217.75	130.7
		***3.65***	***19.54***	***43.75***	***66.24***
Parkland	67.53	53.28	40.01	22.14	8.69
		***21.1***	***40.75***	***67.21***	***87.13***
Boreal	29.88	26.85	14.1	12.13	9.63
		***10.14***	***52.81***	***59.4***	***67.77***
Rocky Mountain	8.76	8.73	7.47	6.09	0.81
		***0***	***24.32***	***66.49***	***78.38***
Foothills	1.85	1.85	1.4	0.62	0.4
		***0.34***	***14.73***	***30.48***	***90.75***
Total provincial grasslands	495.13	463.68	374.44	258.73	150.23
		***6.35***	***24.38***	***47.75***	***69.66***

Remaining grasslands area (ha) per Natural Region and in total province for the status-quo model, SQ, and four progressive grassland conversion scenarios, S2–S5. Numbers in bold italics indicate the percent of grassland converted to cropland for each grassland conversion scenario (S2–S5), compared to the status-quo model, SQ scenario.

The numerical comparison between the six Natural Regions in Alberta identified three distinct patterns of change in current density across conversion scenarios (Table 2). Specifically: (1) the Grassland and Parkland regions exhibited a dramatic loss in mean normalized current density across scenarios (SQ to S5); (2) the Foothills and Rocky Mountain regions showed an overall increase in both, mean normalized current density values and the percent change in current density as conversion scenarios progressed from SQ to S5; and (3) the Boreal and Canadian Shield regions experienced little or no change.

**Fig 4:**
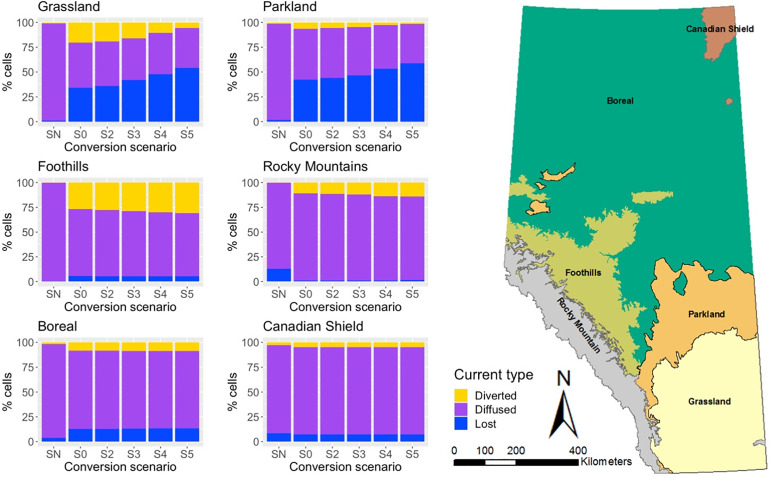
Quantification of current type changes per Natural Region. Quantification of the change in current type (blue: lost current, purple: diffused current, yellow: diverted current) represented as percent of map cells in each current type in the 6 Natural Regions of Alberta across grassland conversion scenarios. Arrows indicate directions of diverted current. NM is the null model where current density was modelled based on resistance due to topography and water bodies only. SQ is the status-quo model where current density was modelled based on resistance due to topography, water bodies, and the average degree of anthropogenic modifications in 2014, and S2–S5 are the modeled scenarios resulting from grassland conversion based on the LSRS classes 2–5 into cropland.

These patterns of change in numerical values were also reflected in the proportions of map cells classified as lost, diffused, or diverted current (Fig 4). The Grassland and Parkland Natural Regions showed a progressive increase in the proportion of lost current, while the proportion of diverted current decreased. This indicates that these landscapes became increasingly impermeable across scenarios. Conversely, the Foothills and Rocky Mountain regions exhibited a continued increase in the proportion of diverted current, highlighting their growing importance for maintaining large-scale connectivity across the province. The Boreal and Canadian Shield regions showed a negligible effect of scenarios S2–S5 compared to the SQ model (Fig 4), with the majority of map cells classified as diffused current. S1 Table includes a detailed report of map cell percentages per current density type in each Natural Region for all grassland conversion scenarios.

In contrast to historical changes, the simulated changes in mean normalized current density between SQ and S2 were minimal, as most grasslands on soils with slight limitations for crop cultivation had already been converted (SQ). As the conversion scenarios progressed from S2 to S5, the remaining grasslands in southeastern municipalities experienced a continuous decline in mean normalized current density. Meanwhile, current was increasingly diverted to the west and northeast of the province through municipalities in the Foothills, the Rocky Mountain, and Boreal regions.

At the quarter-section scale, the map of percent change in normalized current density provided more detail (Fig 3, b) and reflected the current flow patterns identified at the 100 m grid resolution (Fig 2). In the Grassland region, the simulated changes from SQ to S2 were concentrated at the northern, western, and southwestern edges of the region, where it borders the Parkland, Foothills, and Rocky Mountain regions. High rates of change also occurred throughout the Parkland region but did not extend significantly into the Boreal region. These changes became more pronounced with the S3 scenario, including a concentration of current density loss in the southeastern corner of the Grassland region. In the most extreme scenarios (S4 and S5), the patterns of current density loss changed markedly, primarily affecting the eastern part of the province, extending from the Grassland region into the Boreal region.

## Discussion

Taken together, our results highlight how human footprint effects on structural landscape connectivity interact across scales: at a local scale, the current density of a spatial unit depends not only on the resistance of its human footprint but also on the permeability of the surrounding landscape. The spatial configuration of natural or semi-natural landscape features, including natural and cultivated grasslands, thus plays a crucial role in either enabling or impeding ecological flow within a region. At a larger spatial scale, the simulations predicted a diversion of ecological flow from areas that lost much of their permeability (e.g., Parkland and Grassland regions) to other regions (e.g., Foothills).

Our results of reduced structural landscape connectivity in the Parkland and Grassland regions are in concordance with previous studies that deemed these areas as continental and national grassland connectivity bottlenecks [20,21]. Specifically, the pronounced loss of ecological flow in southern Alberta aligns with continental-scale connectivity assessments showing that the northern edge of the Great Plains, where Alberta’s grasslands are located, harbors some of the lowest connectivity values across North America [21]. Additionally, our characterization of the Rocky Mountain and Foothills Natural Regions as major recipients of diverted ecological flow supports the role of these regions as climatic and topographic refugia, as found in studies of large mammal movement corridors [33,35]. Moreover, our results extend on the previous findings by showing how grassland connectivity is likely to be further degraded not just by historical land conversion, but also by future agricultural expansion into marginal lands under shifting climate and economic pressures. Unlike most past studies, which have focused on current connectivity or past loss [3,4], our scenario-based approach quantifies potential future declines in connectivity and highlights priority areas where maintaining or restoring grasslands could have important benefits. Furthermore, while some studies have emphasized functional connectivity for specific taxa, our structural connectivity approach offers a scalable framework to assess landscape permeability across jurisdictional boundaries and land management processes, which is essential for multi-species planning and conservation prioritization [12,38].

Here, we discuss the implications of the historic and potential future trends in structural landscape connectivity that we identified for Alberta. We conclude with considerations about the use and limitations of scenario-based modeling of structural landscape connectivity based on the degree of naturalness.

### Impact of present-state human landscape alteration

The structural landscape connectivity maps reveal low connectivity in central and southern Alberta, a pattern also observed at a continental scale along the northern edge of the Great Plains [2,3,21]. A comparison between the NM and SQ current density maps illustrated that the Parkland region, along with the western and northern parts of the Grassland region, have already lost most of their landscape permeability. Therefore, conservation efforts should focus on restoring corridors in these regions. In contrast, the central and southeastern parts of the Grassland region, where the majority of the remnant grasslands are located, have remaining conduits that maintain some level of internal permeability and provide larger-scale connections to grasslands across the Prairie ecozone, spanning provincial and national boundaries. Areas of high current density, mapped based on the degree of naturalness, are supported by actual functional connectivity data [36,38,40], including GPS-tracked movements of caribou, wolves, moose, and elk in western Canada [41]. However, more research is needed to determine whether these remaining connectivity routes are indeed suitable for many species or if unmapped characteristics, such as habitat quality and farming practices, pose additional resistance. For instance, the ability of Pronghorn antelope to pass a fence is highly dependent on clearance height [42].

The SQ current density map identifies high ecological flow pathways in the western and southwestern areas of the province, highlighting the significant role of the transition zone between the Grassland and the Rocky Mountain regions in facilitating large-scale connectivity. Because these areas remain largely natural, they received significant diverted ecological flow in the SQ model as connectivity decreased in the Grassland and Parkland regions. Therefore, it is paramount to maintain this functional corridor to support a wide range of taxa and species across the province. However, since our connectivity models only considered the degree of naturalness, the simulated diversion of ecological flow along the foot of the Rocky Mountain (Foothills and Rocky Mountain regions) may not be realistic for many grassland species for two reasons: First, these corridors are distant from the ecological flow pathways in the southeastern Grassland region, exceeding the movement capabilities of many grassland species. Second, they consist of habitat types that may not be suitable for many grassland species. Historically, mammals like grizzly bears had home ranges extending from the Rocky Mountain into the Grassland region for seasonal feeding and denning, but today, they rarely venture into the Grassland region [43]. Restoring corridors between the existing grasslands in the central and southeastern Grassland region and the transition zone to the Rocky Mountain region could help reintroduce these species to their natural home range.

### Impact of potential future grassland conversion

The S2 scenario, which incorporated remnant grassland patches with more suitable soil and climate conditions for crop farming into historically converted lands, predicted minimal change in structural landscape connectivity compared to the SQ scenario. These typically fragmented grasslands are likely to continue benefiting from safeguards such as designation as Indigenous lands, protected areas, grazing leases, and other public land systems that may have historically prevented their conversion. Continuing these preservative land-use practices is crucial for maintaining current levels of landscape permeability and ecological flow routes, especially in regions where these typically fertile black soils have already been extensively converted. As conversion scenarios incorporated grassland areas with progressively restrictive soil and climate conditions for crop farming (S3–S5 scenarios), it was predicted that the remaining landscape permeability would increasingly diminish, particularly in the Grassland region. The existing grasslands in the southeastern corner of the province are under increasing pressure of losing landscape permeability [3,23,25]. Even without future conversion, the remaining patches of grasslands in this region are highly fragmented [4] and lack the ecological and functional properties associated with intact grasslands [40,41].

In Alberta, there is an ongoing risk associated with the conversion of grasslands for agricultural purposes [23]. However, the majority of unprotected remnant grassland areas in the Grassland region are characterized by marginal brown soils and experience moderate to severe challenges related to water availability. These challenges are expected to worsen due to climate change projections of warmer temperatures and reduced soil water availability for crop farming in southern Alberta [44,45]. As a result, the risk of converting less suitable marginal soils to croplands in the Grassland region is expected to remain low, and any future conversion would likely require adopting alternative irrigation and cropping strategies tailored to the region. In contrast, in the southern Boreal region and parts of the Parkland region, where climatic change is expected to improve conditions for crop farming, coupled with their already higher soil suitability, future conversion of grasslands to agriculture is likely to accelerate. This underscores the need for proactive connectivity planning to maintain landscape permeability and enhance large-scale connections between the Prairie ecozone and the remaining grasslands within and beyond the transition zone to the Boreal ecozone. Given expectations that the Prairie ecozone will likely extend further north [46], such planning is essential to preserve existing connectivity routes in the face of changing climatic conditions.

### Limitations and future directions

While this study provides valuable insights into how progressive grassland conversion may affect structural landscape connectivity in Alberta, several limitations should be considered. First, the modelling framework adopts a species-agnostic approach and quantifies resistance based on the degree of human modification, which, while objective and repeatable, does not incorporate differences in species’ movement capabilities or habitat preferences [20,33]. Second, the model did not distinguish between different grassland habitat types (e.g., native grass vs. tame pasture) or account for habitat quality and management practices, which can significantly influence functional connectivity [12]. In addition, this study does not treat highly modified green spaces like city parks or recreational fields. Although such areas are often overlooked in connectivity assessments, urban green spaces can provide stepping-stone habitats or partial corridors for certain adaptable species, especially in fragmented landscapes [12,47]. Third, the analysis was based on current and past land use and did not explicitly incorporate projected climate change impacts on land suitability or species distributions. Climate-driven changes in soil moisture and vegetation patterns are likely to affect both the extent of future grassland conversion and the functionality of remaining corridors [39]. Incorporating these complexities into future scenario-based modelling efforts (e.g., including species-specific dispersal traits, dynamic climate projections, and finer grassland habitat distinctions) will be essential for supporting more robust connectivity conservation and land-use planning strategies.

## Conclusions

We compared the current structural landscape connectivity status with the connectivity status under progressive grassland conversion scenarios to quantify changes in landscape permeability and ecological flow across scales in Alberta. Interpreting current density values in structural landscape connectivity modelling poses significant challenges, as both low and high values can indicate departure from ideal conditions of diffused current across the landscape. It is clear that the subjectivity involved in selecting spatial resolution, making decisions regarding resistance map construction and calculations, determining the placement of current source points (nodes), and assigning human footprint values of resistance based on expert opinion, may collectively influence the resulting current density maps and divergent trends.

Our findings underscored the detrimental effect of potential future grassland conversion on structural landscape connectivity across Alberta. This highlights the importance of conserving grasslands to preserve connectivity across the province, particularly in regions where they have higher suitability for agriculture. Simulated changes in structural landscape connectivity were most pronounced at the finer spatial scale (i.e., quarter section). These fine-scale patterns of current density helped identify where landscape permeability changes have been concentrated historically and are likely to occur if future grassland conversion takes place. Given that land-use decisions in Alberta are typically made at fine scales (e.g., quarter-sections), integrating the role of these management units is crucial for enhancing landscape permeability and supporting effective grassland conservation and connectivity planning province-wide. Further studies are needed to determine how unmapped factors such as habitat type and quality, as well as land management practices influence the role of quarter-section management units in planning larger-scale structural landscape connectivity for Alberta’s fragmented grasslands. If grassland conversion is allowed to continue, conservation of the remaining connections of ecological flow will play a critical role in ensuring the genetic diversity that will be needed for wildlife populations to adapt to climate change.

## Supporting information

S1 Fig Current density distributions and current type classification based on the null model (NM). Distribution of normalized current density values on a log scale for 6 connectivity modelling scenarios in Alberta. (**SN**) is the null-scenario where current density was modelled based on resistance due to topography and water bodies only (no anthropogenic modifications), (**SQ**) is the status-quo scenario where current density was modelled based on resistance due to topography, water bodies, and the average degree of anthropogenic modifications (degree of physical footprint and intensity of human use), (**S2**) conversion scenario where all grasslands located within LSRS class-2 were converted into cropland, (**S3**) conversion scenario where all grasslands located within LSRS class-2 and 3 were converted into cropland, (**S4**) conversion scenario where all grasslands located within LSRS class 2–4 were converted into cropland, (**S5**) conversion scenario where all grasslands located within LSRS class 2–5 were converted into cropland.
(DOCX)

S1 Table Percent of map cells representing three current density types (diverted, diffused, and low). Classification of current density values was based on the quantiles of the distribution of the null model (NM) as follows: (1) Diverted current (top 5 percentile); (2) Diffused current (middle 90 percentile); and (3) lost or low current (bottom 5 percentile) of current density values in the distribution. See S1 Fig for the distributions of maps under different scenarios.
(DOCX)

S2 Table Descriptive statistics of resistance and current density values. Descriptive statistics of resistance and normalized current density values at the provincial scale of Alberta for the null model, NM, the status-quo model, SQ, and four progressive grassland conversion scenarios, S2–S5, based on the simulated conversion of remaining grasslands in classes 2–5 of the Land Suitability Rating System (LSRS).
(DOCX)

S3 Table Summary statistics of current density by municipality. Mean normalized current density ± standard deviations per municipality in Alberta for the null model, NM, the status-quo model, SQ, and four progressive grassland conversion scenarios, S2–S5, based on the simulated conversion of remaining grasslands in classes 2–5 of the Land Suitability Rating System (LSRS).
(DOCX)
