# Supplementary material for: Divergent trends in structural landscape connectivity from historic and potential future grassland conversion in Alberta, Canada
Source: PLoS One. 2025 Aug 1;20(8):e0325729. doi: 10.1371/journal.pone.0325729 (PMC12316227; doi:10.1371/journal.pone.0325729)
Supplement: S1 Fig — Distribution of normalized current density values on a log scale for 6 connectivity modelling scenarios in Alberta. (SN) is the null-scenario where current density was modelled based on resistance due to topography and water bodies only (no anthropogenic modifications), (SQ) is the status-quo scenario where current density was modelled based on resistance due to topography, water bodies, and the average degree of anthropogenic modifications (degree of physical footprint and intensity of human use), (S2) conversion scenario where all grasslands located within LSRS class-2 were converted into cropland, (S3) conversion scenario where all grasslands located within LSRS class-2 and 3 were converted into cropland, (S4) conversion scenario where all grasslands located within LSRS class 2–4 were converted into cropland, (S5) conversion scenario where all grasslands located within LSRS class 2–5 were converted into cropland. (DOCX) [file pone.0325729.s001.docx]

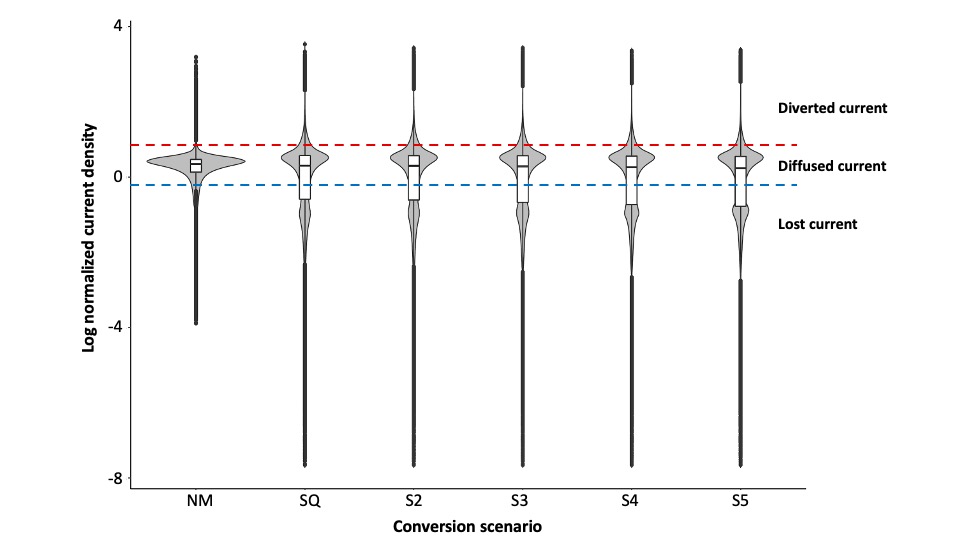


**S1 Fig. Current density distributions and current type classification based on the null model (NM)**. Distribution of normalized current density values on a log scale for 6 connectivity modelling scenarios in Alberta. (**SN**) is the null-scenario where current density was modelled based on resistance due to topography and water bodies only (no anthropogenic modifications), (**SQ**) is the status-quo scenario where current density was modelled based on resistance due to topography, water bodies, and the average degree of anthropogenic modifications (degree of physical footprint and intensity of human use), (**S2**) conversion scenario where all grasslands located within LSRS class-2 were converted into cropland, (**S3**) conversion scenario where all grasslands located within LSRS class-2 and 3 were converted into cropland, (**S4**) conversion scenario where all grasslands located within LSRS class 2–4 were converted into cropland, (**S5**) conversion scenario where all grasslands located within LSRS class 2–5 were converted into cropland.
