# Supplementary material for: Divergent trends in structural landscape connectivity from historic and potential future grassland conversion in Alberta, Canada
Source: PLoS One. 2025 Aug 1;20(8):e0325729. doi: 10.1371/journal.pone.0325729 (PMC12316227; doi:10.1371/journal.pone.0325729)
Supplement: S1 Table — Classification of current density values was based on the quantiles of the distribution of the null model (NM) as follows: (1) Diverted current (top 5 percentile); (2) Diffused current (middle 90 percentile); and (3) lost or low current (bottom 5 percentile) of current density values in the distribution. See S1 Fig for the distributions of maps under different scenarios. (DOCX) [file pone.0325729.s002.docx]

**S1 Table. Percent of map cells representing three current density types (diverted, diffused, and low)**. Classification of current density values was based on the quantiles of the distribution of the null model (NM) as follows: (1) Diverted current (top 5 percentile); (2) Diffused current (middle 90 percentile); and (3) lost or low current (bottom 5 percentile) of current density values in the distribution. See Appendix 1 for the distributions of maps under different scenarios.

| **Scenario** | **Natural region** | **%Low current** | **%Diffused current** | **%Diverted current** |
| --- | --- | --- | --- | --- |
| NM | Boreal | 3.83 | 94.31 | 1.86 |
| NM | Canadian Shield | 8.3 | 88.79 | 2.91 |
| NM | Foothills | 0.4 | 99.48 | 0.11 |
| NM | Grassland | 1.15 | 97.98 | 0.87 |
| NM | Parkland | 1.57 | 97.31 | 1.13 |
| NM | Rocky Mountain | 12.85 | 86.98 | 0.17 |
| SQ | Boreal | 12.74 | 78.86 | 8.4 |
| SQ | Canadian Shield | 7.36 | 87.84 | 4.8 |
| SQ | Foothills | 5.49 | 67.72 | 26.8 |
| SQ | Grassland | 34.02 | 45.65 | 20.33 |
| SQ | Parkland | 42.26 | 51.15 | 6.59 |
| SQ | Rocky Mountain | 0.95 | 88.13 | 10.92 |
| S2 | Boreal | 12.85 | 78.72 | 8.43 |
| S2 | Canadian Shield | 7.35 | 87.85 | 4.8 |
| S2 | Foothills | 5.43 | 66.87 | 27.71 |
| S2 | Grassland | 35.75 | 44.97 | 19.28 |
| S2 | Parkland | 44.01 | 50.38 | 5.61 |
| S2 | Rocky Mountain | 0.94 | 87.7 | 11.36 |
| S3 | Boreal | 13.15 | 78.27 | 8.57 |
| S3 | Canadian Shield | 7.32 | 87.83 | 4.86 |
| S3 | Foothills | 5.42 | 65.71 | 28.87 |
| S3 | Grassland | 41.98 | 42.11 | 15.91 |
| S3 | Parkland | 46.51 | 48.89 | 4.61 |
| S3 | Rocky Mountain | 1.06 | 86.83 | 12.11 |
| S4 | Boreal | 13.28 | 78.09 | 8.63 |
| S4 | Canadian Shield | 7.32 | 87.83 | 4.84 |
| S4 | Foothills | 5.4 | 64.45 | 30.15 |
| S4 | Grassland | 47.8 | 41.64 | 10.56 |
| S4 | Parkland | 53.28 | 44.01 | 2.71 |
| S4 | Rocky Mountain | 1.13 | 85.2 | 13.67 |
| S5 | Boreal | 13.42 | 77.88 | 8.7 |
| S5 | Canadian Shield | 7.32 | 87.88 | 4.8 |
| S5 | Foothills | 5.34 | 63.67 | 30.99 |
| S5 | Grassland | 54.23 | 40.28 | 5.49 |
| S5 | Parkland | 58.69 | 39.77 | 1.55 |
| S5 | Rocky Mountain | 1.44 | 84.6 | 13.96 |
