# Supplementary material for: Divergent trends in structural landscape connectivity from historic and potential future grassland conversion in Alberta, Canada
Source: PLoS One. 2025 Aug 1;20(8):e0325729. doi: 10.1371/journal.pone.0325729 (PMC12316227; doi:10.1371/journal.pone.0325729)
Supplement: S2 Table — Descriptive statistics of resistance and normalized current density values at the provincial scale of Alberta for the null model, NM, the status-quo model, SQ, and four progressive grassland conversion scenarios, S2–S5, based on the simulated conversion of remaining grasslands in classes 2–5 of the Land Suitability Rating System (LSRS). (DOCX) [file pone.0325729.s003.docx]

**S2 Table. Descriptive statistics of resistance and current density values.** Descriptive statistics of resistance and normalized current density values at the provincial scale of Alberta for the null model, NM, the status-quo model, SQ, and four progressive grassland conversion scenarios, S2–S5, based on the simulated conversion of remaining grasslands in classes 2–5 of the Land Suitability Rating System (LSRS).

|  | **Resistance** | | **Normalized current density** | |
| --- | --- | --- | --- | --- |
| **Scenario** | **Range** | **Mean SD** | **Range** | **Mean SD** |
| NM | 1 – 167.9 | 4.8 11.5 | 0.0204 – 24.5 | 1.3 0.4 |
| SQ | 1 – 1089.0 | 23.8 69.1 | 0.0004 – 34.2 | 1.3 0.9 |
| S2 | 1 – 1089.0 | 24.2 69.4 | 0.0004 – 30.9 | 1.3 0.8 |
| S3 | 1 – 1089.0 | 25.2 69.8 | 0.0004 – 31.0 | 1.3 0.9 |
| S4 | 1 – 1089.0 | 26.5 70.3 | 0.0004 – 28.9 | 1.3 0.8 |
| S5 | 1 – 1089.0 | 27. 6 70.6 | 0.0004 – 29.4 | 1.3 0.8 |
