# Supplementary material for: Divergent trends in structural landscape connectivity from historic and potential future grassland conversion in Alberta, Canada
Source: PLoS One. 2025 Aug 1;20(8):e0325729. doi: 10.1371/journal.pone.0325729 (PMC12316227; doi:10.1371/journal.pone.0325729)
Supplement: S3 Table — Mean normalized current density ± standard deviations per municipality in Alberta for the null model, NM, the status-quo model, SQ, and four progressive grassland conversion scenarios, S2–S5, based on the simulated conversion of remaining grasslands in classes 2–5 of the Land Suitability Rating System (LSRS). (DOCX) [file pone.0325729.s004.docx]

**S3 Table. Summary statistics of current density by municipality.** Mean normalized current density ± standard deviations per municipality in Alberta for the null model, NM, the status-quo model, SQ, and four progressive grassland conversion scenarios, S2–S5, based on the simulated conversion of remaining grasslands in classes 2–5 of the Land Suitability Rating System (LSRS).

| **Municipality** | **NM** | **SQ** | **S2** | **S3** | **S4** | **S5** |
| --- | --- | --- | --- | --- | --- | --- |
| Municipal District of Acadia No. 34 | 0.858±0.205 | 1.287±0.801 | 0.855±0.798 | 0.846±0.785 | 0.596±0.573 | 0.654±0.585 |
| Athabasca County | 1.099±0.446 | 1.53±0.784 | 1.091±0.78 | 1.082±0.786 | 1.073±0.784 | 1.068±0.784 |
| County Of Barrhead No. 11 | 0.774±0.376 | 1.377±0.747 | 0.765±0.746 | 0.745±0.736 | 0.753±0.748 | 0.756±0.756 |
| Beaver County | 0.809±0.471 | 1.504±0.865 | 0.793±0.844 | 0.739±0.771 | 0.609±0.575 | 0.57±0.526 |
| Municipal District of Bonnyville No. 87 | 0.93±0.584 | 1.309±0.71 | 0.914±0.698 | 0.867±0.683 | 0.844±0.668 | 0.823±0.656 |
| Camrose County | 0.711±0.454 | 1.426±0.687 | 0.673±0.563 | 0.661±0.551 | 0.619±0.507 | 0.586±0.473 |
| Cardston County | 1.125±0.339 | 1.331±1.02 | 1.003±0.992 | 0.683±0.704 | 0.658±0.684 | 0.609±0.661 |
| Municipal District of Fairview No. 136 | 0.565±0.282 | 1.264±0.539 | 0.568±0.542 | 0.572±0.549 | 0.576±0.553 | 0.581±0.558 |
| Flagstaff County | 0.742±0.361 | 1.523±0.731 | 0.727±0.719 | 0.711±0.654 | 0.601±0.49 | 0.576±0.459 |
| Municipal District of Foothills No. 31 | 1.195±0.381 | 1.345±1.075 | 1.168±1.08 | 1.062±1.038 | 0.848±0.861 | 0.74±0.711 |
| County Of Forty Mile No. 8 | 1.143±0.341 | 1.386±1.114 | 1.148±1.121 | 1.213±1.323 | 0.839±0.938 | 0.668±0.677 |
| County Of Grande Prairie No. 1 | 0.751±0.333 | 1.336±0.705 | 0.755±0.708 | 0.752±0.707 | 0.752±0.708 | 0.751±0.708 |
| I.D. No. 4 (Waterton) | 1.54±0.331 | 0.787±0.899 | 1.592±0.937 | 1.88±1.198 | 1.984±1.266 | 2.112±1.353 |
| I.D. No. 9 (Banff) | 1.354±0.359 | 0.68±0.814 | 1.365±0.823 | 1.385±0.839 | 1.45±0.886 | 1.525±0.943 |
| I.D. No. 12 (Jasper National Park) | 1.059±0.342 | 0.68±0.549 | 1.066±0.554 | 1.079±0.562 | 1.102±0.578 | 1.121±0.59 |
| I.D. No. 13 (Elk Island) | 1.995±0.661 | 1.192±1.32 | 1.975±1.334 | 1.894±1.419 | 1.822±1.364 | 1.738±1.298 |
| I.D. No. 24 (Wood Buffalo) | 1.674±0.617 | 1.489±0.698 | 1.675±0.698 | 1.678±0.7 | 1.678±0.699 | 1.678±0.699 |
| Kneehill County | 0.541±0.279 | 1.43±0.567 | 0.52±0.537 | 0.459±0.406 | 0.456±0.39 | 0.459±0.373 |
| Lac Ste. Anne County | 0.787±0.417 | 1.338±0.714 | 0.768±0.703 | 0.741±0.681 | 0.754±0.697 | 0.758±0.707 |
| Lacombe County | 0.614±0.445 | 1.461±0.597 | 0.589±0.509 | 0.565±0.467 | 0.552±0.45 | 0.514±0.4 |
| Lamont County | 0.724±0.379 | 1.661±0.722 | 0.683±0.674 | 0.646±0.596 | 0.6±0.556 | 0.579±0.537 |
| Leduc County | 0.631±0.362 | 1.524±0.566 | 0.595±0.535 | 0.587±0.525 | 0.587±0.525 | 0.579±0.52 |
| Lethbridge County | 0.584±0.336 | 1.564±0.543 | 0.585±0.544 | 0.554±0.504 | 0.519±0.417 | 0.525±0.421 |
| County Of Minburn No. 27 | 0.787±0.413 | 1.392±0.769 | 0.741±0.713 | 0.699±0.638 | 0.629±0.575 | 0.568±0.492 |
| Mountain View County | 0.844±0.318 | 1.539±0.926 | 0.839±0.934 | 0.775±0.9 | 0.727±0.831 | 0.696±0.777 |
| County Of Newell | 1.263±0.41 | 1.49±1.102 | 1.247±1.088 | 1.182±1.042 | 1.097±1.117 | 0.852±0.861 |
| County Of Paintearth No. 18 | 1.152±0.407 | 1.499±1.228 | 1.132±1.226 | 1.166±1.262 | 0.84±0.824 | 0.78±0.767 |
| Parkland County | 0.728±0.48 | 1.346±0.669 | 0.704±0.65 | 0.678±0.627 | 0.682±0.639 | 0.677±0.641 |
| Municipal District of Peace No. 135 | 0.635±0.36 | 1.22±0.589 | 0.63±0.587 | 0.63±0.59 | 0.636±0.595 | 0.642±0.601 |
| Municipal District of Pincher Creek No. 9 | 1.527±0.387 | 1.155±1.082 | 1.467±1.079 | 1.271±1.045 | 1.232±1.012 | 1.161±1.05 |
| Ponoka County | 0.703±0.354 | 1.478±0.681 | 0.669±0.642 | 0.648±0.617 | 0.627±0.595 | 0.586±0.541 |
| Municipal District of Provost No. 52 | 1.334±0.351 | 1.293±1.561 | 1.242±1.526 | 1.125±1.451 | 0.974±1.2 | 0.787±0.803 |
| Red Deer County | 0.596±0.394 | 1.508±0.54 | 0.598±0.541 | 0.568±0.502 | 0.557±0.486 | 0.515±0.434 |
| Rocky View County | 0.985±0.381 | 1.49±1.058 | 0.96±1.058 | 0.904±1.033 | 0.731±0.847 | 0.662±0.685 |
| Smoky Lake County | 0.941±0.408 | 1.348±0.699 | 0.916±0.677 | 0.887±0.676 | 0.846±0.64 | 0.819±0.618 |
| Municipal District of Smoky River No. 130 | 0.648±0.377 | 1.56±0.786 | 0.652±0.791 | 0.657±0.796 | 0.666±0.808 | 0.673±0.809 |
| Municipal District of Spirit River No. 133 | 0.464±0.237 | 1.432±0.381 | 0.462±0.378 | 0.466±0.382 | 0.466±0.376 | 0.469±0.379 |
| County Of St. Paul No. 19 | 0.766±0.458 | 1.257±0.625 | 0.738±0.601 | 0.694±0.566 | 0.651±0.525 | 0.616±0.495 |
| Starland County | 0.733±0.284 | 1.379±0.676 | 0.723±0.679 | 0.633±0.567 | 0.619±0.507 | 0.556±0.444 |
| County Of Stettler No. 6 | 0.93±0.465 | 1.452±0.982 | 0.873±0.904 | 0.804±0.814 | 0.772±0.763 | 0.649±0.619 |
| Strathcona County | 0.652±0.46 | 1.405±0.649 | 0.611±0.599 | 0.606±0.599 | 0.591±0.59 | 0.561±0.559 |
| Sturgeon County | 0.55±0.33 | 1.594±0.534 | 0.527±0.502 | 0.514±0.478 | 0.507±0.461 | 0.495±0.449 |
| Municipal District of Taber | 0.805±0.323 | 1.505±0.771 | 0.8±0.765 | 0.809±0.773 | 0.697±0.685 | 0.685±0.634 |
| Thorhild County | 0.821±0.325 | 1.659±0.717 | 0.809±0.707 | 0.794±0.702 | 0.78±0.695 | 0.765±0.691 |
| County Of Two Hills No. 21 | 0.8±0.414 | 1.287±0.706 | 0.761±0.674 | 0.723±0.629 | 0.66±0.57 | 0.613±0.523 |
| County Of Vermilion River | 0.938±0.341 | 1.266±0.837 | 0.844±0.729 | 0.8±0.684 | 0.743±0.626 | 0.675±0.559 |
| Vulcan County | 0.684±0.318 | 1.48±0.679 | 0.675±0.671 | 0.572±0.58 | 0.489±0.39 | 0.491±0.359 |
| Municipal District of Wainwright No. 61 | 1.152±0.325 | 1.292±1.04 | 1.074±0.957 | 1.013±0.899 | 0.899±0.812 | 0.722±0.691 |
| County Of Warner No. 5 | 0.882±0.309 | 1.433±0.843 | 0.845±0.818 | 0.725±0.74 | 0.599±0.534 | 0.552±0.445 |
| Westlock County | 0.67±0.343 | 1.612±0.64 | 0.657±0.629 | 0.647±0.617 | 0.647±0.614 | 0.644±0.61 |
| County Of Wetaskiwin No. 10 | 0.718±0.446 | 1.468±0.651 | 0.678±0.614 | 0.663±0.61 | 0.658±0.609 | 0.638±0.593 |
| Wheatland County | 0.635±0.326 | 1.472±0.713 | 0.601±0.664 | 0.507±0.564 | 0.476±0.432 | 0.479±0.41 |
| Municipal District of Willow Creek No. 26 | 1.051±0.449 | 1.43±0.919 | 0.981±0.837 | 0.691±0.606 | 0.598±0.469 | 0.522±0.352 |
| Municipality Of Crowsnest Pass | 1.483±0.296 | 0.941±0.745 | 1.562±0.798 | 1.691±0.889 | 1.849±1.012 | 2.027±1.137 |
| Kananaskis Improvement District | 1.614±0.249 | 0.684±0.849 | 1.624±0.858 | 1.645±0.871 | 1.785±0.939 | 1.847±0.965 |
| Cypress County | 1.904±0.281 | 1.286±1.259 | 1.886±1.262 | 1.691±1.226 | 1.381±1.217 | 0.984±0.991 |
| Clearwater County | 1.6±0.453 | 1.036±1.004 | 1.614±1.016 | 1.621±1.026 | 1.649±1.041 | 1.664±1.048 |
| Municipal District of Bighorn No. 8 | 1.891±0.324 | 0.855±1.04 | 1.909±1.053 | 1.926±1.066 | 2.033±1.15 | 1.959±1.12 |
| Brazeau County | 1.385±0.338 | 1.424±1.082 | 1.392±1.098 | 1.38±1.11 | 1.386±1.115 | 1.389±1.12 |
| Municipality Of Jasper | 1.152±0.368 | 0.781±0.519 | 1.16±0.524 | 1.173±0.532 | 1.197±0.546 | 1.217±0.557 |
| Special Areas No. 3 | 1.479±0.265 | 1.347±1.281 | 1.465±1.268 | 1.415±1.252 | 1.097±1.23 | 0.901±1.017 |
| Special Areas No. 4 | 1.506±0.31 | 1.34±1.357 | 1.468±1.362 | 1.322±1.391 | 1.168±1.25 | 0.946±1.141 |
| I.D. No. 25 (Willmore Wilderness) | 0.99±0.275 | 0.721±0.366 | 0.995±0.368 | 1.003±0.371 | 1.019±0.377 | 1.031±0.382 |
| Woodlands County | 1.904±0.29 | 1.293±1.158 | 1.931±1.177 | 1.956±1.203 | 1.983±1.225 | 2.013±1.248 |
| Municipal District of Greenview No. 16 | 1.34±0.344 | 1.186±0.73 | 1.349±0.737 | 1.36±0.748 | 1.378±0.762 | 1.391±0.773 |
| Yellowhead County | 1.579±0.353 | 1.181±0.934 | 1.595±0.947 | 1.61±0.96 | 1.631±0.98 | 1.646±0.992 |
| Northern Sunrise County | 1.411±0.366 | 1.401±0.587 | 1.414±0.588 | 1.416±0.591 | 1.421±0.593 | 1.424±0.594 |
| Municipal District of Ranchland No. 66 | 1.613±0.259 | 0.843±0.683 | 1.605±0.67 | 1.68±0.732 | 1.653±0.791 | 1.609±0.861 |
| Birch Hills County | 0.819±0.295 | 1.367±0.722 | 0.821±0.725 | 0.825±0.732 | 0.831±0.74 | 0.839±0.748 |
| Saddle Hills County | 1.12±0.282 | 1.313±0.888 | 1.125±0.893 | 1.116±0.887 | 1.123±0.894 | 1.13±0.902 |
| Clear Hills County | 1.136±0.273 | 1.284±0.542 | 1.139±0.544 | 1.139±0.545 | 1.145±0.548 | 1.151±0.552 |
| Mackenzie County | 1.408±0.35 | 1.381±0.507 | 1.409±0.508 | 1.411±0.511 | 1.413±0.512 | 1.413±0.513 |
| Big Lakes County | 1.468±0.47 | 1.198±1.007 | 1.481±1.017 | 1.498±1.034 | 1.519±1.051 | 1.534±1.062 |
| District of Lesser Slave River No. 124 | 1.697±0.488 | 1.353±0.958 | 1.707±0.966 | 1.725±0.984 | 1.745±1 | 1.766±1.016 |
| Regional Municipality of Wood Buffalo | 1.441±0.487 | 1.337±0.617 | 1.441±0.617 | 1.444±0.618 | 1.442±0.617 | 1.442±0.617 |
| County Of Northern Lights | 1.255±0.285 | 1.346±0.59 | 1.257±0.591 | 1.256±0.598 | 1.259±0.599 | 1.261±0.6 |
| Municipal District of Opportunity No. 17 | 1.595±0.432 | 1.461±0.589 | 1.596±0.589 | 1.602±0.592 | 1.605±0.594 | 1.608±0.596 |
| Lac La Biche County | 1.277±0.509 | 1.325±0.678 | 1.273±0.676 | 1.265±0.681 | 1.258±0.679 | 1.254±0.679 |
| Special Areas No. 2 | 1.722±0.338 | 1.435±1.351 | 1.675±1.319 | 1.61±1.351 | 1.418±1.381 | 1.061±1.183 |
| I.D. No. 349 | 1.448±0.363 | 1.396±0.412 | 1.444±0.412 | 1.438±0.415 | 1.429±0.414 | 1.424±0.414 |
